# Multiplex allele-specific polymerase chain reaction-based prevalence study of canine trapped neutrophil syndrome in Thailand

**DOI:** 10.14202/vetworld.2023.2271-2276

**Published:** 2023-11-12

**Authors:** Chommanad Lerdkrai, Nuch Phungphosop

**Affiliations:** Department of Physiology, Faculty of Veterinary Medicine, Kasetsart University, Bangkok, Thailand

**Keywords:** Border Collie, multiplex allele specific-polymerase chain reaction assay, Thai Ridgeback, trapped neutrophil syndrome, *vacuolar protein sorting 13 homolog B* genotype

## Abstract

**Background and Aim:**

Trapped neutrophil syndrome (TNS) is an autosomal recessive genetic disorder found in Border Collies and is characterized by peripheral neutropenia and myeloid hyperplasia of bone marrow. The underlying cause of TNS is associated with a 4-base pair deletion mutation in the *vacuolar protein sorting 13 homolog B* (*VPS13B*) gene. In this study, we proposed and validated a novel multiplex allele specific-polymerase chain reaction (MAS-PCR) technique to assess the prevalence of TNS using *VPS13B* genotypes of Border Collies and Thai Ridgebacks in Thailand.

**Materials and Methods:**

We assessed the prevalence of TNS in 100 Border Collies and 30 Thai Ridgebacks using MAS-PCR-based allelic discrimination technique of the *VPS13B* gene. We then confirmed the *VPS13B* genotypes by direct DNA sequencing.

**Results:**

A total of 130 samples were successfully genotyped using MAS-PCR assays. Of the two dog breeds examined, the *VPS13B* mutation was present in Border Collies, whereas Thai Ridgebacks were unaffected by this mutation. In Border Collies, 96% of dogs tested had an intact *VPS13B* genotype, whereas the remaining individuals had a heterozygous mutation genotype, with prevalence and mutated *VPS13B* allele frequencies of 4% and 2%, respectively.

**Conclusion:**

Using a novel MAS-PCR assay targeting the *VPS13B* gene, this study demonstrates for the first time that carriers of TNS exist in Border Collies in Thailand. This assay is a reliable and cost-effective tool for diagnosing TNS based on *VPS13B* genotypes and is suitable for routine clinical practice.

## Introduction

Trapped neutrophil syndrome (TNS) is a rare autosomal recessive disorder that was first reported in purebred Border Collies in New Zealand [1] and Australia [2]. To date, confirmed cases of TNS have been reported in the UK [3], Japan [4], Israel [5], and the USA [6, 7]. In 2011, Shearman and Wilton [2] discovered the causative mutation underlying TNS by performing linkage analysis using microsatellite markers. This study discovered a 4-base pair (bp) deletion mutation within exon 19 of the *vacuolar protein sorting 13 homolog B* (*VPS13B*) gene (g.4411956_4411960delGTTT) on canine chromosome 13. This mutation leads to a premature termination codon and a truncated protein product in dogs with a clinical indication of TNS [2]. The *VPS13B* gene encodes transmembrane proteins that are involved in diverse cellular processes, including vesicular trafficking, endosomal/lysosomal transport, protein sorting [8], and the maintenance of the Golgi apparatus structure [9]. Furthermore, VPS13B proteins play important roles in developing and functioning of various systems, including the ocular system, central nervous system, and hematopoiesis system [8, 10]. Interestingly, the genetic basis of canine TNS mimics a rare human genetic disease known as Cohen syndrome, which is also associated with mutations in the *VPS13B* gene [2].

Trapped neutrophil syndrome is characterized by specific clinicopathological features, including peripheral neutropenia and myeloid hyperplasia of bone marrow [1]. The most common clinical manifestations are developmental delay, lethargy, anorexia, pyrexia, vomiting, diarrhea, elongated narrow muzzle, joint swelling, lameness, and lymphadenopathy [1, 11]. Despite the identification of a causal genetic mutation, the precise mechanisms responsible for the symptoms and features of TNS remain unknown. The diagnosis of TNS is based on clinical examinations and histological evaluation of bone marrow, combined with genetic testing to confirm the presence of homozygous mutations in the *VPS13B* gene [2]. There is currently no specific and approved therapeutic regimen for TNS; however, antibiotics and immunosuppressive medications can be used to control symptoms and prevent secondary infections [3, 4, 6, 12]. Importantly, the prognosis for long-term survival is guarded to poor, as affected animals are highly susceptible to secondary infections and early death [1, 2].

Border Collies have gained popularity in Thailand over the last few decades. However, there is relatively limited awareness and knowledge of breed-specific disorders. The actual prevalence of TNS remains unknown because *VPS13B* genotypes in Border Collies and native Thai breeds are rarely tested. To the best of our knowledge, no previous studies have been conducted in Thailand to investigate the presence of TNS and/or the distribution of the *VPS13B* genotype.

Thus, the present study aimed to develop and validate a novel multiplex allele-specific polymerase chain reaction (MAS-PCR) protocol for determining the mutation status of the *VPS13B* gene. In addition, we estimated the prevalence of TNS based on *VPS13B* genotyping assay data from a population of Border Collies and Thai Ridgebacks.

## Materials and Methods

### Ethical approval

This study was conducted in accordance with the guiding principles of the Institutional Animal Care and Use Committee of Kasetsart University (Ethics Approval No. ACKU65-VET-049).

### Study period and location

The study was conducted from July 2022 to June 2023 at the Department of Physiology, Faculty of Veterinary Medicine, Kasetsart University, Thailand.

### Animals and sample collection

A total of 130 peripheral blood-ethylenediaminetetraacetic acid samples were collected from clinically healthy dogs living in Central, Northern, and Southern Thailand. These dogs ranged from 4 months to 8 years of age, and their *VPS13B* genotype statuses had not been previously investigated. Data for 100 Border Collies and 30 Thai Ridgebacks were collected from private dog owners and breeding kennels, with or without pedigree information, and with informed consent. This sample included 30 private dog owners, four Border Collie breeding kennels, and two Thai Ridgeback breeding kennels.

### *Vacuolar protein sorting 13 homolog B* genotyping through multiplex allele-specific PCR

For our PCR genotyping protocol, genomic DNA was first extracted from blood samples using a QIAmp DNA Blood Mini Kit (Qiagen GmbH, Germany), with all procedures following the manufacturer’s protocol. Extracted DNA was then stored at −20°C until further use. In this study, we designed new oligonucleotide primers using the Primer-Blast tool function from the National Center for Biotechnology Information (NCBI) (https://blast.ncbi.nlm.nih.gov/blast.cgi) (Table-1) to amplify the target region of the *VPS13B* gene. Wild-type and mutated canine *VPS13B* reference sequences were obtained from GenBank using accession numbers CP050604.1 and HM036106.1, respectively [2].

**Table-1:** Primer sequences used in the MAS-PCR assay for characterization of the *VPS13B* genotypes.

Primer name	Nucleotide sequence 5’ to 3’
F1	TTTTATGCTCCCACCAGCAG
F2	CTTATATAACTGGCTT**GTTT**ATCAG
F3	CCCAGTCTTATATAACTGGCTTATC
R1	CAGTGTTTCGCTTTACCAGC

A 4-bp deletion mutation in the *VPS13B* locus is marked in bold. MAS-PCR=Multiplex allele-specific polymerase chain reaction, *VPS13B*=*Vacuolar protein sorting 13 homolog B*

**Figure-1:**
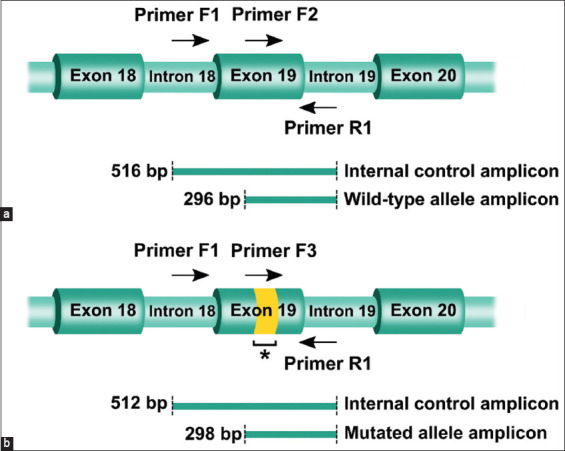
Schematic diagram illustrating the binding sites of primers used in the multiplex allele-specific polymerase chain reaction (PCR) assay to detect a 4-base pair (bp) deletion mutation within exon 19 of the *vacuolar protein sorting 13 homolog B* (*VPS13B*) gene. (a) The primer set PCR1 used for the amplification of a 296-bp wild-type allele-specific amplicon (F2 and R1) and a 516-bp internal control amplicon (F1 and R1). (b) The primer set PCR2 used for the amplification of a 298-bp mutated allele-specific amplicon (F3 and R1) and a 512-bp internal control amplicon (F1 and R1). The horizontal arrows indicate the region and direction of the primers. The asterisk (*) indicates a mutated region of exon 19.

For the MAS-PCR assay, we designed outermost primers, a forward internal control primer (F1) and a common reverse primer (R1), to amplify an internal control fragment encompassing the mutated region of exon 19. In addition, we developed allele-specific primers, including a forward wild-type allele-specific primer (F2) and a forward mutated allele-specific primer (F3), for use in conjunction with the outermost primers. As shown in Figures-1a and b, the primer set PCR 1, which was used to detect the wild-type *VPS13B* allele, consisted of a three-primer combination mixture (F1, F2, and R1). This amplified a 296-bp amplicon specific to the wild-type allele and a 516-bp internal control amplicon. The primer set PCR 2 was similar to PCR 1, except that it used the F3 primer instead of F2 primer. This yielded a 298-bp amplicon that was specific to the mutated allele and a 512-bp internal control amplicon.

Amplification of the *VPS13B* gene by MAS-PCR assay was performed for each sample in a two-tube format. The first tube contained a reaction mixture with a total volume of 25 μL for detection of the wild-type allele. This consisted of 1X Platinum^®^ *Taq* PCR buffer (10X, Invitrogen, USA), 0.2 mM of each dNTP (Biotechrabbit, Germany), 1.75 mM MgCl_2_ (Invitrogen), 0.3 μM each of F1 and R1 primers (Biobasic Inc., Canada), 0.2 μM F2 primer (Biobasic Inc.), 1 U Platinum^®^ *Taq* DNA Polymerase (Invitrogen), and 50−80 ng of genomic DNA. A second tube contained the PCR mixture for the detection of the mutated allele. The contents of this tube were similar to the first, except that the F2 primer was replaced with the F3 primer. Polymerase chain reaction amplification was performed using a MiniAmp Plus Thermal Cycler (Thermo Fisher Scientific, USA). The reaction procedure for amplification of both wild-type and mutated alleles consisted of an initial denaturation step at 95°C for 5 min; followed by 35 cycles of denaturation at 95°C for 30 s, annealing at 60°C for 90 s, and extension at 72°C for 1 min, with a final extension step at 72°C for 10 min. Subsequently, the amplified products were electrophoresed on a 1.5% agarose gel containing ethidium bromide, and visualized under ultraviolet illumination.

### Sequencing

To confirm the *VPS13B* genotype, all samples were subjected to direct DNA sequencing. The *VPS13B* gene segment encompassing the mutation site was amplified using the F1 and R1 primers. The PCR reaction mixture and amplification protocol were similar to those used for the MAS-PCR assay, with the exception that annealing at 58°C for 2 min was used. Amplified products were then purified using a NucleoSpin^®^ Gel and PCR Clean-up Purification Kit (Macherey-Nagel, Germany) according to the manufacturer’s protocol. DNA was then sequenced by Macrogen Inc. (Korea) using the F1 primer. Sequence analysis was performed using the SnapGene^®^ Viewer program (SnapGene^®^ Software, GSL Biotech LLC, USA). Finally, sequence similarity comparisons were determined using the NCBI database (https://blast.ncbi.nlm.nih.gov/blast.cgi).

## Results

**Figure-2:**
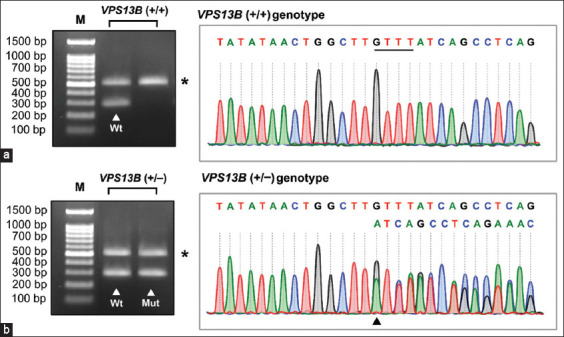
Agarose gel electrophoresis images of multiplex allele-specific polymerase chain reaction products and DNA sequencing confirmation of mutation at the *vacuolar protein sorting 13 homolog B* (*VPS13B*) locus (a) The fragment pattern corresponding to the *VPS13B* (+/+) and the *VPS13B* (+/–) genotype. The lanes marked by white arrowheads represent the 296-base pair (bp) wild-type allele- and 298-bp mutated allele-specific amplicons. The asterisk (*) displayed in each panel indicates the approximately 512- or 516-bp internal control amplicons. Lane M, 100-bp DNA Marker. (b) Alignment of representative sequences of *VPS13B* (+/+) genotype (upper) and *VPS13B* (+/–) genotype (lower). The black underlined sequence refers to a 4-bp deletion mutation in the *VPS13B* target region. The black arrowhead indicates the overlapping traces of wild-type and mutated *VPS13B* alleles.

The genotypic patterns obtained using the newly developed MAS-PCR assay for the *VPS13B* gene included both *VPS13B* (+/+) and *VPS13B* (+/–) genotypes. Agarose gel electrophoresis images and electropherograms for each genotype are shown in Figures-2a and b, respectively. In this assay, the *VPS13B* (+/+) genotype showed coamplification of a 516-bp internal control amplicon and a single 296-bp wild-type allele-specific amplicon, whereas the *VPS13B* (+/–) genotype showed the simultaneous detection of two allele-specific amplicons, consisting of a 298-bp mutant allele- and a 296-bp wild-type allele-specific amplicon, as well as a 512- or 516-bp internal control amplicon. After optimization, the internal control and allele-specific amplicons were successfully amplified with distinguishable products and similar intensities in all samples evaluated. In addition, the results obtained using this assay demonstrated 100% concordance with direct DNA sequencing.

The results of *VPS13B* genotyping and the allele frequencies of the 130 dogs are summarized in Table-2. Of the two dog breeds examined in this study, the mutation in the *VPS13B* gene was only found in Border Collies. In 100 Border collies examined, the *VPS13B* (+/+) genotype was predominant, accounting for 96%, while the remaining individuals had the *VPS13B* (+/–) genotype, accounting for 4%. No homozygous individuals for the mutated allele were observed in this population. The mutant *VPS13B* allele frequency was 2% in Border Collies. In Thai Ridgebacks; however, all dogs tested showed an intact *VPS13B* (+/+) genotype.

**Table-2:** Genotype and allele frequencies of the VPS13B gene in 130 dogs.

Breed	N	Genotype frequencies			Allelic frequencies (%)	
	
		*VPS13B* (+/+) n (%)	*VPS13B* (+/–) n (%)	*VPS13B* (–/–) n (%)	Wild-type allele	Mutated allele
Border Collie	100	96 (96)	4 (4)	0 (0)	98	2
Thai Ridgeback	30	30 (100)	0 (0)	0 (0)	100	0

*VPS13B* (+/+)=Homozygous wild-type genotype, VPS13B (+/–)=Heterozygous mutation genotype, *VPS13B* (–/–)=Homozygous mutation genotype, N=Total number of dogs tested for each breed, n=Number of dogs for each genotypic pattern, *VPS13B*=*Vacuolar protein sorting 13 homolog B*

## Discussion

Several genetic testing techniques have been developed to identify *VPS13B* genotypes since the discovery of a mutation in the *VPS13B* gene suggested an association with TNS [2]. These methods include a conventional PCR-based length polymorphism analysis with microchip electrophoresis [4] and a real-time PCR assay using TaqMan minor groove binder probes [13]. These assays provide rapid and robust diagnostic tools for determining *VPS13B* mutation status; however, these methods are relatively expensive and require special laboratory equipment. In this study, we propose an accurate and cost-effective multiplex PCR protocol combined with allele-specific amplification as an alternative screening test for identifying *VPS13B* genotypes. In this system, two allele-specific forward primers, consisting of the F2 and F3 primers, together with a reverse R1 primer, were used to amplify a 296-bp amplicon specific for the wild-type allele and a 298-bp amplicon specific for the mutated allele. To ensure the accuracy and reliability of amplification in each reaction, a 512- or 516-bp internal control product obtained from a forward F1 primer and a reverse R1 primer was included in each assay. Our results showed that this protocol was able to achieve efficient amplification of all expected product sizes using identical PCR settings. Furthermore, all amplified products, including both allele-specific and internal control amplicons, appeared as distinct bands on the gel image, allowing us to identify the genotype at the *VPS13B* locus (Figure-2a).

After establishing this protocol, we used it to investigate the genotype status of the *VPS13B* gene in a population of Border Collies and Thai Ridgebacks in Thailand. The genotyping results were confirmed by direct DNA sequencing (Table-2). We found that the proposed assay was successful in identifying different *VPS13B* genotypes with high accuracy and that these results were completely consistent with the genotypic data obtained by sequencing. This assay is suitable for clinical testing due to its simplicity and the use of basic genetic laboratory tools. Nevertheless, a possible limitation of using our technique is that it may be difficult to distinguish between two allele-specific products due to their similar size, leading to false-negative or false-positive errors. Therefore, both positive and negative controls must be incorporated into each experiment to ensure the accuracy and reliability of the results obtained using this technique.

In 2011, Shearman and Wilton [2] reported a pioneering study on the distribution of canine *VPS13B* gene mutations in several countries. In a survey of 1436 Border Collies from six European countries, including Norway, Finland, Czech Republic, Germany, the Netherlands, and the UK, the carrier frequencies of TNS were approximately 8.1%–13%. No affected individuals were identified in this survey, other than in Norway, where one case of *VPS13B* (–/–) genotype was detected, which represented 1.2% of the sample. The overall mutant allele frequencies of *VPS13B* gene ranged from 4.1%–6.8% in these regions [2]. Furthermore, this study also revealed that the TNS was present in Australia and the USA. Among the 260 Border Collies tested in Australia, the carrier frequency was 15.4%, and the mutant allele frequency was 7.7%. A similar trend was observed in a population of 315 individuals investigated in the USA, where carrier and mutant allele frequencies were 16.5% and 8.3%, respectively. These results were slightly higher than those reported in the European region [2]. Besides these countries, two studies have also reported the presence of the *VPS13B* mutation in Border Collies in Japan. Shearman and Wilton [2] discovered a carrier frequency of 16.9% and a mutant allele frequency of 8.4% in a sample of 83 Border Collies. However, with a larger sample size of 441 individuals, Mizukami *et al*. [13] reported a slightly lower carrier frequency of 11.1% and a mutant allele frequency of 5.5%. As in Australia and the USA, none of the cases described in both studies harbored a *VPS13B* (–/–) genotype [2, 13].

In our present study of 100 Border Collies, we included 26 privately owned dogs with unrelated parentages and 74 dogs that were obtained from four breeding kennels in Thailand. Among these kennels, 47 dogs originated from kennel A, and approximately one-third of this population shared the same parental lineage. The remaining individuals were obtained evenly from kennels B, C, and D, and were of unrelated parentage across kennels. Moreover, approximately half of all dogs evaluated in this survey had descended from dog populations in the USA, while the remainder originated from Australia and Europe, including France, Hungary, Italy, and the UK. Our investigation demonstrated that the majority of dogs tested had an intact *VPS13B* genotype (96%), and none of the cases studied were homozygous for the focal *VPS13B* mutation. Four individuals in kennel C, all of whom were associated with the same maternal line, were identified as carriers of TNS. In this survey, the carrier frequency (4%) and mutant allele frequency (2%) were relatively low compared to previous investigations in Australia, the USA, Europe [2], and Japan [2, 13]. Based on the previous studies and the present survey, it is clear that *VPS13B* genotypic assays reveal that the prevalence of TNS in Border Collies is relatively low in many different countries. In addition to the results published here, we recently investigated the prevalence of two additional gene mutations, including *multi-drug resistance 1* (*MDR1*) and *non-homologous end-joining factor 1* (*NHEJ1*) genes in several dog breeds in Thailand [14, 15]. In the sample of 45 Border Collies examined for these surveys, the mutated *MDR1* allele was not observed in any of the dogs tested, indicating a remarkably low prevalence of the *MDR1* gene mutation in this breed [14]. Furthermore, the prevalence of the *NHEJ1* gene mutation associated with Collie eye anomaly was relatively low in this population, as indicated by a carrier frequency of 15.6%, and a mutant allele frequency of 7.8% [15]. Taken together, our findings therefore provide compelling evidence that the risk of Thai Border Collies developing these common inherited genetic disorders is relatively low.

In addition to the Border Collie breed, we investigated the distribution of *VPS13B* genotypes in Thai Ridgebacks. As a native hunting dog breed, Thai Ridgebacks have distinct genetic characteristics and are not descended from the Collie lineage. To the best of our knowledge, no previous studies have investigated the genotypic pattern of the *VPS13B* gene in this breed. In this study, the majority of dogs tested most likely descended from unrelated parents. In addition, approximately two-thirds of all individuals were obtained evenly from two breeding kennels, and the remaining one-third were privately owned. As expected, no mutations in the *VPS13B* gene were identified in any of the 30 dogs evaluated, indicating that this native breed has little or no incidence of TNS. Interestingly, a similar finding has been reported by previous genotypic surveys of the *MDR1* and *NHEJ1* genes in Thailand [14, 15]. Although the population of Thai Ridgebacks in these studies was relatively small, our findings provide valuable evidence that this native breed has a relatively healthy genetic profile and is not susceptible to genetic disorders commonly associated with sheepdog lineages.

## Conclusion

The novel MAS-PCR assay developed in this study represents a reliable and efficient tool for discriminating normal individuals from TNS carriers in the studied populations. Accurate identification of TNS carriers enables breeders to improve their breeding strategies and decrease the likelihood of producing carriers or affected offspring. Nevertheless, the limitations of this study should also be emphasized. One limitation is the relatively small sample size used in the study, which may have affected the accuracy of the results. Further studies with a larger sample size and diverse populations would strengthen our findings and provide more reliable estimates of the mutation rate at the *VPS13B* locus and its association with TNS in the studied dog breeds. Another limitation is the limited availability of pedigree data for the evaluated dogs, which contributes to biased results. Therefore, comprehensive pedigree data are required for future studies to assess parental relationships and improve interpretation of results.

## Authors’ Contributions

CL: Examined the clinical condition of the animals, collected blood samples, designed the study, performed the experiments, analyzed data, and drafted, reviewed, and edited the manuscript. NP: Provided technical laboratory support. Both authors have read, reviewed, and approved the final manuscript.
